# Integrated Omics Reveals the Orchestrating Role of Calycosin in Danggui Buxue Tang, a Herbal Formula Containing Angelicae Sinensis Radix and Astragali Radix, in Inducing Osteoblastic Differentiation and Proliferation

**DOI:** 10.3389/fphar.2021.670947

**Published:** 2021-06-23

**Authors:** Kenneth K L Kwan, Tin Yan Wong, Anna X D Yu, Tina T X Dong, Henry H N Lam, Karl W K Tsim

**Affiliations:** ^1^ Shenzhen Key Laboratory of Edible and Medicinal Bioresources, Shenzhen Research Institute, Shenzhen, China; ^2^ Division of Life Science and Center for Chinese Medicine, The Hong Kong University of Science and Technology, Shenzhen, China; ^3^ Department of Chemical and Biological Engineering, The Hong Kong University of Science and Technology, Kowloon, China

**Keywords:** mesenchymal stem cells, multi-omics, bone differentiation, herbal materials, mass-spectrometry

## Abstract

Systems biology unravels the black box of signaling pathway of cells; but which has not been extensively applied to reveal the mechanistic synergy of a herbal formula. The therapeutic efficacies of a herbal formula having multi-target, multi-function and multi-pathway are the niches of traditional Chinese medicine (TCM). Here, we reported an integrated omics approach, coupled with the knockout of an active compound, to measure the regulation of cellular signaling, as to reveal the landscape in cultured rat osteoblasts having synergistic pharmacological efficacy of Danggui Buxue Tang (DBT), a Chinese herbal formula containing Angelicae Sinensis Radix and Astragali Radix. The changes in signaling pathways responsible for energy metabolism, RNA metabolism and protein metabolism showed distinct features between DBT and calycosin-depleted DBT. Here, our results show that calycosin within DBT can orchestrate the osteoblastic functions and signaling pathways of the entire herbal formula. This finding reveals the harmony of herbal medicine in pharmacological functions, as well as the design of drug/herbal medicine formulation. The integration of systems biology can provide novel and essential insights into the synergistic property of a herbal formula, which is a key in modernizing TCM.

## Introduction

The metaphor of “butterfly effect” is based on chaos theory and encapsulates the concept to suggest a small change at any one point in a complex system resulting in dominant effects happening elsewhere (Dodd, 2011). This “butterfly effect” is accounting for the multi-functional properties of a complex formula of traditional Chinese medicine (TCM), whereby several specific variables initially may have minor effects, and thereafter which have a significant impact on the therapeutic efficacy of final product (Xu et al., 2013). Indeed, reports have provided cues for chemical ingredients of herbal formula in orchestrating the pharmaceutical effects (Niemeyer et al., 2013; Gong et al., 2015).

**GRAPHICAL ABSTRACT:**
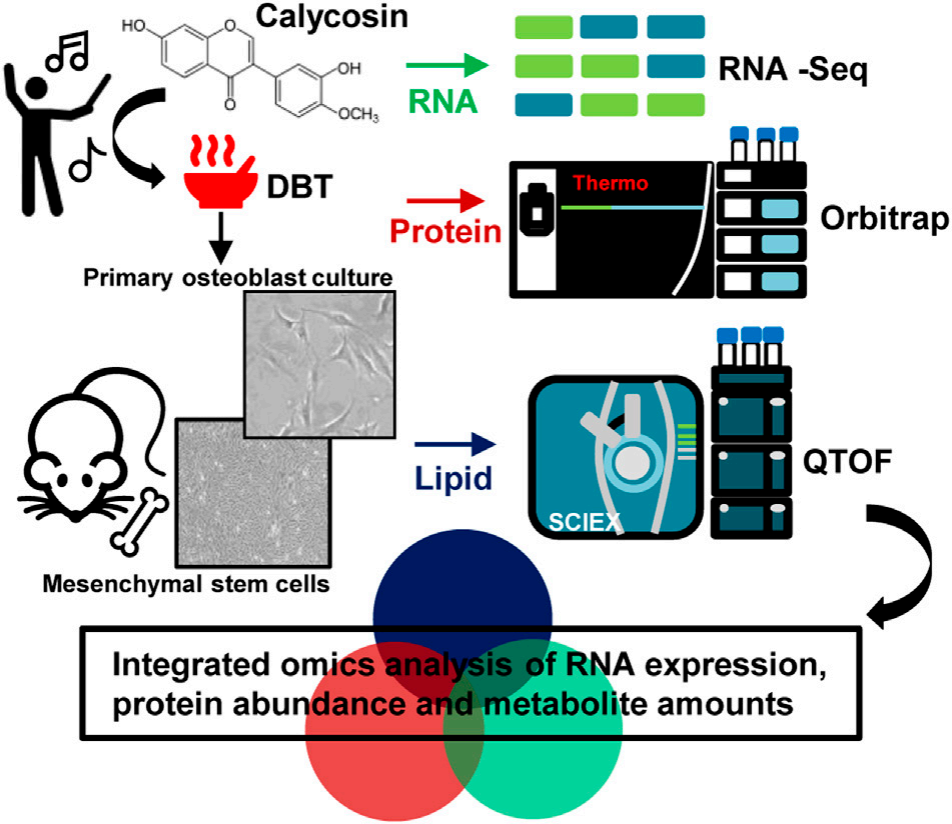


Among those TCM formula, Danggui Buxue Tang is one of the simplest formulae, containing two herbs: Angelicae Sinensis Radix (ASR; roots of *Angelica sinensis* Oliv) and Astragali Radix [AR; roots of *Astragalus membranaceus* (Fisch). Bunge or *A. membranaceus* (Fisch). Bunge var. *mongholicus* (Bunge) Hsiao] at the ratio of 1:5 (Dong et al., 2006; Zheng et al., 2010), recorded in “*Neiwaishang Bianhuo Lun*” by *Li Dongyuan* in Jin dynasty (about AD 1247). This ancient herbal formula was suggested to be consumed by patients suffering from “Blood” and “Qi” deficiency syndromes (Gong A. G. W. et al., 2016). Nowadays, DBT is suggested to be taken every day as a remedy for menopause, i.e., recovery bone fracture in aged women (Gao et al., 2007; Lin et al., 2017). Although this TCM formula has been used for over 800 years; the mechanism of its pharmacological efficacy is still not known. In accord to the basic principles of TCM, the synergistic property and compatibility of different compounds are proposed to account for the action mechanism of herbal formula. However, the correlation between active chemicals within an herbal decoction and the detailed underlying mechanisms have not been elucidated, and which hinders the acceptance of TCM by the general public.

In analyzing the action mechanism of DBT, we have studied different roles of an individual component within the herbal extract; however, none of them can act like functionally as that in an herbal formula (Kwan et al., 2019). DBT stimulated the growth of bone, or osteoblast, in cell and animal models, when compared to herbal extract deriving from AR or ASR (Choi et al., 2011; Zhou et al., 2018). Accounting for better pharmacological efficacy of herbal formula, the concentrations of crucial DBT’s ingredients were found to be more soluble in water when they were prepared by boiling two herbs together (Dong et al., 2006). This evidence could be an outcome of synergism resulting from physicochemical interaction within the complex herbal formula. Calycosin, a phytoestrogen, is a major active flavonoid in AR and, therefore, as well as in DBT (Gong et al., 2015). Calycosin was proposed to be a distinct chemical in orchestrating the pharmacological function of DBT. In line with this notion, the calycosin-depleted DBT decoction (DBT_Δcal_) showed insignificant activity in inducing osteoblastic differentiation, as compared with that of parental DBT (Gong et al., 2015). Interestingly, calycosin alone did not show activities as in DBT, suggesting a key synergistic role of calycosin playing with those chemicals within DBT decoction.

To reveal mechanistic signaling of DBT in osteoblastic differentiation, we employed transcriptomics and metabolomics as tools to identify and quantify transcript and metabolite in DBT-treated osteoblasts. The DBT-triggered signaling pathways and metabolites were compared between authentic DBT and DBT_Δcal_. The identified biomolecules, regulated by the herbal decoction in cell culture, could serve as biomarkers in quality control of the herbal decoction, and subsequently, those identified biomolecules could provide prosperous detection window in solving the mechanism of multi-components of TCM.

## Materials and methods

### Preparation and Quality Control of Herbal Medicines

All source of chemical or consumable were provided by Sigma-Aldrich. The preparation of herbal extract of DBT was described before (Song et al., 2004; Zheng et al., 2010; Gong et al., 2015; Gong A. et al., 2016; Gong A. G. W. et al., 2016). Roots of 3 year-old *A. membranaceus* var. *mongholicus* (AR) from Shanxi Province and 2 year-old *A. sinensis* roots (ASR) from Minxian of Gansu Province were utilized. Authentication of herbal medicine was examined by Dr. Tina Dong (herbalist) in Hong Kong University of Science and Technology (HKUST) based on the Standards of Hong Kong Materia Medica. The voucher specimens (voucher # 02-9-1 for ASR and voucher # 02-10-4 for AR) were stored in Centre for Chinese Medicine of HKUST. To prepare the herbal extract, DBT (numbers of AR and ASR in a weight ratio of 5:1) was extracted in eight volumes of H_2_O (v/w) at boiling point for 2 h. Finally, this process was done two times. The pooled formula was dehydrated under vacuum. Identification and quantification of active compounds of DBT were conducted on an Agilent (high-performance liquid chromatography) HPLC system (Agilent, Waldbronn, Germany), coupled with a diode array detector (DAD) and an evaporative light scattering detector (ELSD). The herbal extracts were isolated by an Agilent C_18_ column (1.8 μm, 50 mm × 4.6 mm). The mobile phase contained of 0.1% FA in ACN (B) and 0.1% FA in H_2_O (A). HPLC setting was as below: 0–2 min, 20-20% (B); 2–7 min, 20–34% (B); 7–12 min, 34-34% (B); 12–16 min, 34–65% (B); 16–18 min, 65–80% (B). The solvent velocity was optimized at 0.3 ml/min. The eluate was introduced into DAD and ELSD for further analysis. To prepare DBT_Δcal_, a semi-preparative C_18_ column (10.0 mm × 250 mm, 5 μm) was utilized to deplete calycosin from DBT. The detail of preparation of DBT_Δcal_ was mentioned previously (Gong et al., 2015). The DBT_Δcal_ was examined by HPLC-DAD-ELSD system. The eluate, obtained by the chemical-depletion method, was dried with nitrogen and resuspended in H_2_O for biological experiments, as well as analytical measurements.

### Rat Osteoblast Culture and Micromass Culture

Animal protocols had been revised and approved by The Animal Experimentation Ethics Committee of Hong Kong University of Science and Technology and Department of Health, Hong Kong (No. 17-283 for Animal Ethics Approval), under the instructions of “Principles of Laboratory Animal Care” (NIH publication No. DH/HA&P/8/2/3). The postnatal day 1 SD rat was dissected to obtain calvaries. Tissues were digested by 1% trypsin for 10 min, 0.2% collagenase for 20 min and 0.2% collagenase for the another 45 min, respectively (Yu et al., 2020). Afterwards, the supernatant was obtained via centrifugation at 1,500 rpm for 5 min. Osteoblasts were incubated in MEM-α, supplemented with 10% FBS and 1% PS. Cell proliferation of osteoblast was conducted by 3-(4,5-dimethylthiazol-2-yl)- 2,5-diphenyltetra- zolium bromide (MTT) assay. In rat micromass culture, rat embryos were utilized to cultivate mesenchymal progenitor cells from its limb buds. The ectoderms were removed after enzymatic digestion of limb buds with 0.1% trypsin and 2.4 U of dispase ll for 20 min. The cell number was adjusted to 25 × 10^6^ cells/mL. The micromass was maintained with the CMRL 1066 medium, 10% FBS and 1% PS. The medium was replaced with fresh medium after 24 h in culture and renewed every two days. For histological staining of micromass, the cultures were fixed with 4% paraformaldehyde for 15 min. Next, the cultures were incubated in Alcian blue 8 GS solution (0.1 mg/ml) for 1 h. The cultures were then washed and immersed with glycerol, for Alizarin Red S staining. The fixed cells were stained with 40 mM Alizarin Red S (pH 4.2) for 15 min and immersed with glycerol.

### Alkaline Phosphatase Assay

ALP was extracted in lysis buffer (10 mM HEPES, pH 7.5, 150 mM NaCl, and 0.5% Triton X-100). ALP enzymatic reaction was measured by mixing the cell lysate protein with 10 mM *p*-nitrophenyl phosphate (as a substrate in enzymatic reaction) in a buffer (pH 10.4) containing 0.1 M glycine, 1 mM MgCl_2_, and 1 mM ZnCl_2_ at 37°C, and colorimetric analysis was conducted at 405 nm (Yu et al., 2020).

### Adhesion Assay

Osteoblasts were cultured for 72 h with drug treatment. Cells were trypsinized for 1–2 min and cultured in fresh cultured medium. The cell suspension was then counted to a cell concentration of 4 × 10^5^ cells/mL, and cell suspension was placed to cell culture plate. The cells were cultured for 30 min for cell attachment, and the suspended cells were collected. The unattached cells were counted via phase-contrast microscopy.

### Intracellular Collagen Quantification

Osteoblasts were cultured for 72 h with drug treatment. The cells were incubated in methanol at –20 °C overnight, washed two times with 1X PBS, and placed in 0.1% picrosirius red staining solution (100 μl/well) for 3 h. Next, the cells were washed three times, with 0.1% acetic acid. The staining was recognized by microscope.

### Luciferase Assay

The DNA construct of pRunx2-Luc was obtained from SwitchGear Genomics (Menlo Park, CA). Transfection was conducted via jetPRIME® reagent (Polyplus Transfection). Luciferase assay was conducted via Pierce™ Firefly Luciferase Glow Assay Kit (Thermo Fisher Scientific). The luciferase was extracted from osteoblasts by 0.1 M phosphate buffer (pH 7.8), 0.2% Triton X-100 and 1 mM dithiothreitol (DTT). The chemical luminescent was analyzed by a luminometer.

### Shotgun Proteomics

For protein extraction, osteoblasts were repeated freeze-thaw cycles three times and sonication in 8 M urea buffer (0.1% SDS, pH = 7.4) for 5 min. Next, the cell lysate was precipitated in cold acetone. The treated proteins were resuspended in 4 M urea (pH = 6.5). Forty μg of proteins was reduced by DTT and alkylated by iodoacetamide (IAA). The modified proteins were then cleaved by trypsin (1: 50 w/w) for 18 h at 37 °C. Next, the peptide sample was desalted by C_18_ ZipTip (Millipore, Darmstadt, Germany) and dried by vacuum. Peptides were dissolved in 0.1% FA and were directly loaded onto a C_18_ capillary column (75 μm × 25 cm; 2 μm, 100 Å). The solvent elution was optimized using an Ultimate nanoLC system (Thermo Fisher Scientific, CA) at a flow speed of 300 nl/min, and 120 min LC gradient of 2–90% ACN in 0.1% FA was utilized to isolate peptides. The eluted peptides were detected by Orbitrap Fusion Lumos mass spectrometer (Thermo Fisher Scientific, CA). The electrospray ionization voltage was set at +2.3 kV, and the ion transfer tube temperature was set at 300°C. MS machine setting was as following: one microscan for MS^1^ scan at 60 K resolution, MS^2^ at 30 K resolution. Full MS mass range was: m/z 400–1,500 and MS/MS mass range: m/z 100–2,000. Automatic gain control targeting for MS^2^ was 40 K; maximum injection time was 20 ms; Higher-energy C-trap dissociation energy was 35%, and dynamic exclusion duration was 4 s.

### Protein Identification and Quantification

MS data were analyzed via Thermo Scientific™ Proteome Discoverer™ 2.2. MS^2^ data was searched with SEQUEST® HT against a database of *Rattus norvegicus* Uniport database (UP000002494). Carbamidomethylation (+57.021 Da) of cysteine residues was set as a fixed modification. Oxidation of methionine residues (+15.9949 Da) and acetylation of the protein N-terminus (+42.0106 Da) were considered as variable modifications. MS^1^ tolerance was set as 20 ppm, and MS^2^ was set as 0.8 Da tolerances. Peptide spectral matches were validated using the percolator algorithm, based on *q*-values at a 1% FDR. Next, the proteomics data was conducted using Rt-Aligner and feature mapper nodes, created for untargeted label-free quantification workflow in Proteome Discoverer.

### Lipidomics Analysis

The cellular lipids were obtained by homogenizing 1 × 10^7^ of osteoblasts with 300 μl of LC-MS water and 600 μl of methanol and 450 μl of chloroform. Next, the herbal was facilitated water-organic layer separation via centrifugation at 12,000 g for 15 min. The lower layer was obtained and dried under the nitrogen. Aliquots of 60 μl from lipid samples were pooled for QC sample. The cellular lipids were measured on a Waters Acquity ultra performance liquid chromatography (UPLC) connected with a high-resolution mass spectrometer (TripleTOF 4,600, AB SCIEX). The cellular lipids were analyzed on a Waters UPLC Acquity instrument coupled to a high-resolution MS (TripleTOF 4,600, AB SCIEX). Water C_18_ column (1.7 μm, 2.1 × 100 mm) was utilized for lipid separation. The final acquisition methods were illustrated as following. The mobile phase for lipidomics, A = ACN/water (60:40) with 10 mM NH_4_HCO_3_ and 0.1% FA, B = isopropanol/ACN (90/10) with 10 mM NH_4_HCO_3_ and 0.1% FA. The solvent gradient was 0–10 min: 0–20% B; 10–10.1 min: 20–80% B; 10.1–13 min: 80% B; 13–13.1 min: 80-0% B; 13.1–20 min: 0% B. The solvent speed was optimized at 0.2 ml/min. The MS conditions were set as follows: ion source gas one was 45, ion source gas 2 was 45, curtain gas was 30, temperature was 450 C, ion-spray voltage floating was ±5 kV, de-clustering potential (DP) was 100 V, and collision energy (CE) was 10 V. For TOF-MS scan, the accumulation time was set as 0.1 s per spectra, and TOF masses were acquired from 200 to 1,200 Da. For product ion experiment, the accumulation time was 0.05 s per spectra, and masses were acquired from 100 to 1,160 Da, DP was set at 100 V, and CE was 35 V with ±15 V collision energy spread. Information-dependent acquisition (IDA) experiment was selected to perform MS/MS scan, and its parameters were set as following: exclude isotopes within 4 Da, mass tolerance was 10 ppm, the maximum number of candidate ions per cycle was 20, “for” and “after” were chosen in “exclude former target ions” part and were set at “for 15 s after 2 occurrences” (the time was usually set at half-length of a signal). In addition, “dynamic background subtracts” was chosen in IDA advance module.

### Data Extraction and Processing

Chromatographic peak identification and alignment were performed using Progenesis QI 2.3 (Nonlinear Dynamics, Newcastle Upon Tyne, United Kingdom). Those unstable metabolites were filtered out by applying a cut-off on the coefficient of variation >30% in QC samples. Matrix of normalized ion abundance was exported to SIMCA® (version 13, Umetrics AB) for multivariate data analysis. The potential candidates were selected from S-plots of Orthogonal partial least squares discriminant analysis (OPLS-DA). The biomarkers were further identified with mass fragmentation and matched with Human Metabolome Databases, Kyoto Encyclopedia of Genes and Genomes, METLIN, LipidMap (www.lipidmaps.org), as well as LipidBlast based on their mass fragmentation, retention time and mass accuracy.

### Transcriptomics Analysis

Total RNAs of treated osteoblasts were extracted by RNAzol reagent (Invitrogen). Total RNAs were submitted to RNA-seq analysis (BGI Shenzhen, China) to examine the differential RNA expression in osteoblasts. Quantification analysis was calculated using Fragment Pre kilobase transcriptome per million reads (FPKM). The RNAs with FPKM >1 was used in follow analysis. To limit our attention to differentially expression RNAs, only RNA with fold change higher or lower than ±2 folds (*p* < 0.05) was statistically considered.

### Pathway and Statistical Analysis

Pathway analysis was conducted using Ingenuity Pathway Analysis (QIAGEN). All Multivariate analysis of the sample was conducted using SIMCA®. All data were represented as mean ± standard error of mean (SEM). Statistical analysis was performed with student t-test and Dunnett’s test (SPSS, version 13). Statistically, the difference was classified as significant: *p < 0.05, **p < 0.01 and ***p < 0.001.

## Results

### Calycosin Orchestrates Osteoblastic Function in DBT

**FIGURE 1:**
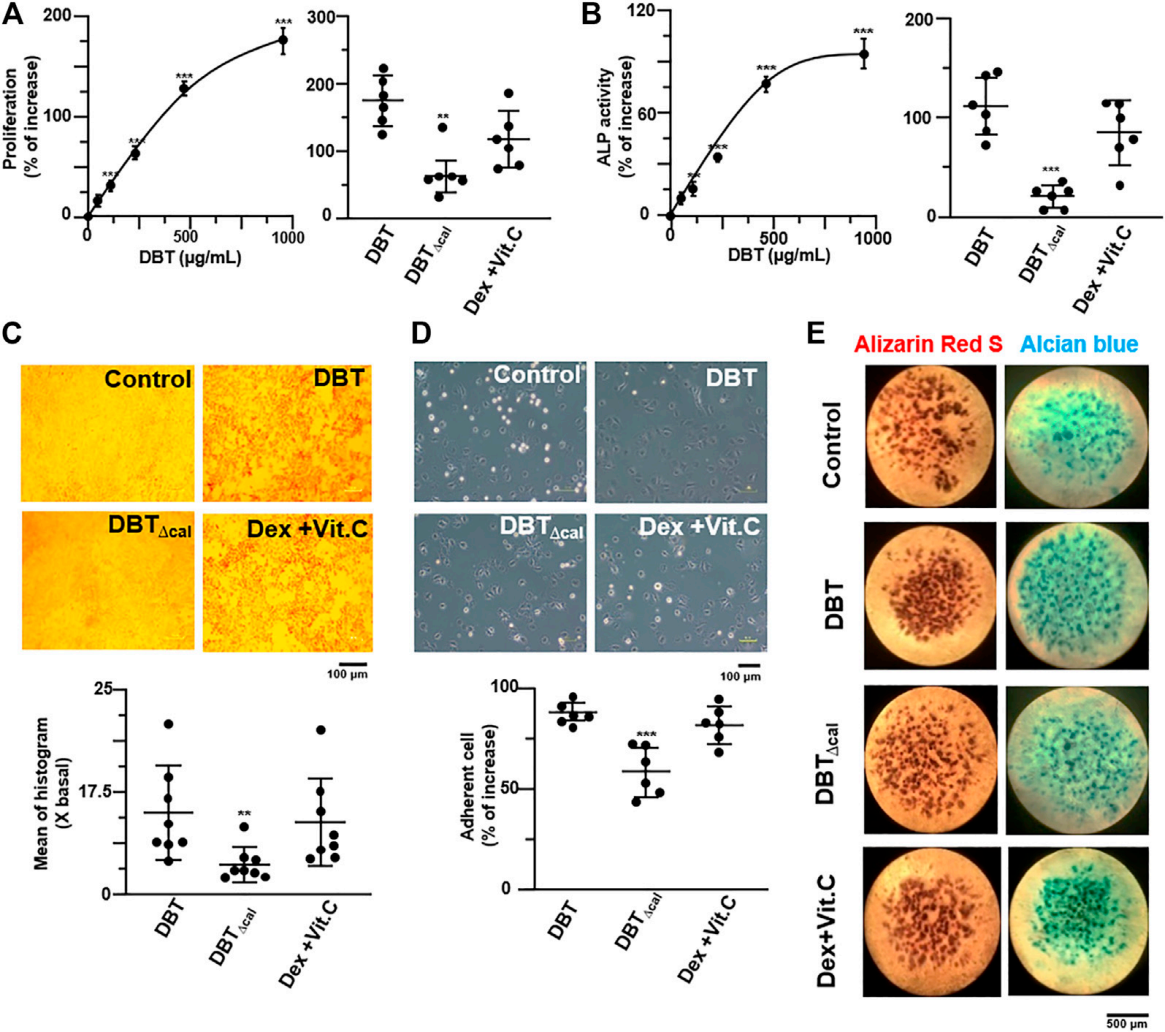
DBT and DBT_∆cal_ in osteoblastic differentiation. **(A)**: DBT at different concentrations was applied onto cultured osteoblast for 7 days to analyze cell proliferation, triggered by the herbal extract. The proliferation was compared between DBT and DBT_∆cal_ (both at 1 mg/ml). **(B):** ALP activity was assayed in different treatments, as in **(A)**. **(C):** The osteoblasts were treated with DBT and DBT_∆cal_ as in **(A)**, before staining with Sirius Red. Micrographs were taken by phase-contrast microscopy (upper panel). One representative result is shown. The quantification of colorimetric intensity was calculated by the image J (lower panel). **(D)**: The treatments of osteoblasts were the same as in **(A)**. The cell adhesion ability was measured (upper panel). The quantification was calculated by the image J (lower panel). **(E)**: Micromass cultures of mouse limb bud cells were incubated in DBT and DBT_∆cal_ (both at 1 mg/ml) for 7 days. The cartilage formed in an inner tissue core, followed by a ring of mineralization near the periphery, as shown by Alcian blue and Alizarin Red S. Vitamin C (250 μM) and dexamethasone (20 nM) served as a positive control (Yu et al., 2020). Values were expressed as % of increase, or fold of change (X basal), of the control in Mean ± SEM, where *n* = 6. *p < 0.05, **p < 0.01, ***p < 0.001.

The herbal extracts at different conditions were prepared by optimized methods (Song et al., 2004), and which were chemically standardized by HPLC. The herbal extracts of DBT (authentic decoction) and DBT_∆cal_ (calycosin-depleted decoction) were subjected to HPLC spectrum by using UV and ELSD detectors: both herbal extracts showed identity except the absence of calycosin (Supplementary Figure S1C,D). In addition, the amounts of major chemicals within the two decoctions were measured (Supplementary Figure S1E). The detail of herbal preparation, as well as their detail characterization, was illustrated in previous reports (Gong et al., 2015; Gong A. et al., 2016; Gong A. G. W. et al., 2016). DBT and DBT_∆cal_ had similar amounts of chemicals, except calycosin: DBT_∆cal_ showed ∼98% depletion of calycosin, as compared to DBT. These chemical analyses served as parameters for repeatability of the below experiments. In cultured osteoblasts, calycosin by itself did not trigger any osteoblastic proliferation, differentiation, as well as RUNX2 promoter, by any means of statistically significant (Supplementary Figure S1B). In DBT preparation, the amount of calycosin was about 0.67 μg in 1 mg of dried DBT herbal extract: this amount showed no effect on the bone cells. However, application of DBT induced cell proliferation, as well as the differentiation biomarker ALP, in a dose-dependent manner (Figures 1A,B). In both scenarios, the DBT-induced osteoblastic growth and differentiation were markedly higher than that of herbal extract from DBT_∆cal_ at same concentration, suggesting an uniqueness of a complete herbal formulation of DBT. To detect the amount of collagen being deposited in osteoblasts, Sirius Red stain was used (Siddiqui and Arshad, 2014). In cultured osteoblasts, applied DBT was able to enhance in the collagen density significantly, at least ∼7-fold, as indicated by dark red clusters of collagens evenly distributed throughout the stimulated region (Figure 1C). Again, the DBT effect was more robust than DBT_∆cal_. After treatment with DBT for 7 days, cultured osteoblasts were gathered and developed their extracellular matrix, i.e., increase adhesion to culture plate: this adhesion property was much less significantly in DBT_∆cal_ herbal extract (Figure 1D). The role of DBT was demonstrated in an *in vivo* model of bone micromass. In DBT-treated micromass, mesenchymal stem cells were stained with Alcian blue (showing overall proteoglycan content) and Alizarin Red S (showing calcium deposit). Applied DBT increased the staining of both dyes (Figure 1E). In contrast, the herbal extract from DBT_∆cal_ showed induction of chondrocyte and osteogenic differentiation; but which was much less, as compared to DBT. In comparison to authentic DBT, the depletion of calycosin in DBT, i.e., DBT_∆cal_, showed weak induction on osteoblastic differentiation in all parameters, suggesting a distinct role of calycosin in DBT function.

### Transcriptomics Analysis

**FIGURE 2:**
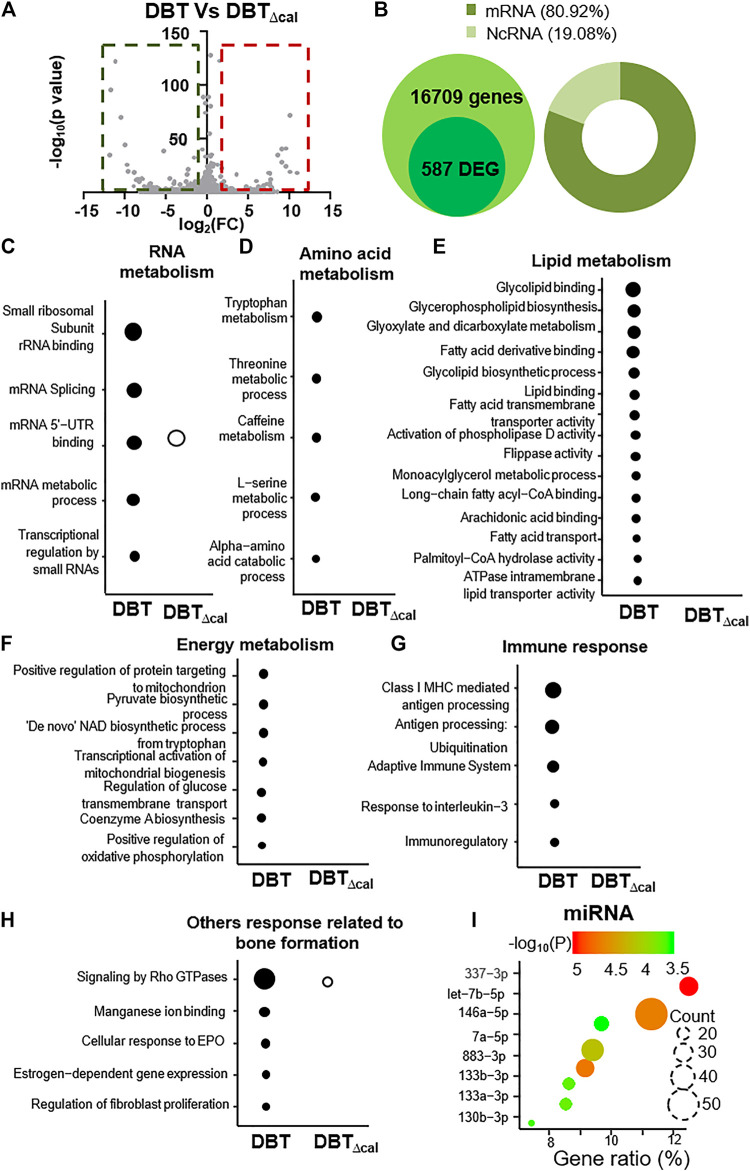
Comparison of transcriptomics between DBT and DBT_∆cal_. The treatment of osteoblasts was as that in Figure 1. **(A):** In RNA-seq results for DBT and DBT_∆cal_, DEGs are deemed significant for *p* < 0.05 and fold change higher/lower than ±1.5, corresponding to the rectangular regions. **(B):** The statistical summary of total detected genes: DEG, mRNA, and ncRNA. **(C–H):** KOBAS analysis enriches the signaling pathway in DEGs via KEGG, Reactome, BioCye, PANTHER, and GO. The size of the circle represents–log_10_(P). **(I):** The miRNA analysis from DEGs. Gene ratio is the ratio of DEG number in corresponding to specific miRNA change.

RNA-seq was utilized to explore the signaling mechanism in osteoblast under treatments of DBT and DBT_∆cal_. Here, we aimed to reveal specific functional role(s) of calycosin in a complex herbal decoction. About 16,709 expressed genes were analyzed by RNA-seq. Besides, we counted the differentially expressed genes (DEGs) having *p*-value < 0.05 of *t*-test, as well as the fold of change, higher/lower than ±1.5 folds. The “volcano plot” of fold change against *p*-value for RNA-seq result was generated. As a result, 587 RNA (240 up regulated and 347 down regulated) were differentially expressed in DBT vs DBT_∆cal_ (Figure 2A). Besides, 80.92% mRNA and 19.08% non-coding RNA were identified to have change in DEGs (Figure 2B). The signaling pathways, induced by different herbal extracts, were determined by KOBAS 3.0. The treatment of DBT induced up regulation of several pathways, in contrasting to DBT_∆cal_, was identified, e.g., small ribosomal subunit rRNA binding (*p* = 9.8 × 10^−4^) and mRNA splicing (*p* = 3 × 10^−3^), having relationship with RNA metabolism (Figure 2C). Meanwhile, the metabolism of amino acid (Figure 2D) and lipid (Figure 2E) were up regulated. The energy metabolism, e.g., transcriptional activation of mitochondrial biogenesis (*p* = 3 × 10^−2^) and positive regulation of oxidative phosphorylation (*p* = 3 × 10^−2^), was also up-regulated (Figure 2F). In parallel, the immune response was induced by increases of transcripts encoding class I MHC mediated antigen processing and presentation (*p* = 3 × 10^−3^) and response to interleukin-3 (*p* = 3 × 10^−2^) (Figure 2G). In addition, the pathways directly related to bone function were identified to be up regulated, e.g., signaling by Rho GTPases (*p* = 2 × 10^−4^) and estrogen-dependent gene expression (*p* = 3.7 × 10^−2^) (Figure 2H). In analysis of miRNA profiling, increased expression of 337-3p and let-7b-5p were identified after DBT treatment (Figure 2I). In line to osteogenic function of DBT, 337-3p and let-7b-5p are playing positive role in bone formation (Geng et al., 2020). The changed pathways, induced by DBT, however were not revealed in the herbal treatment of DBT_∆cal_ (Figures 2C–I).

### Proteomics Analysis

**FIGURE 3:**
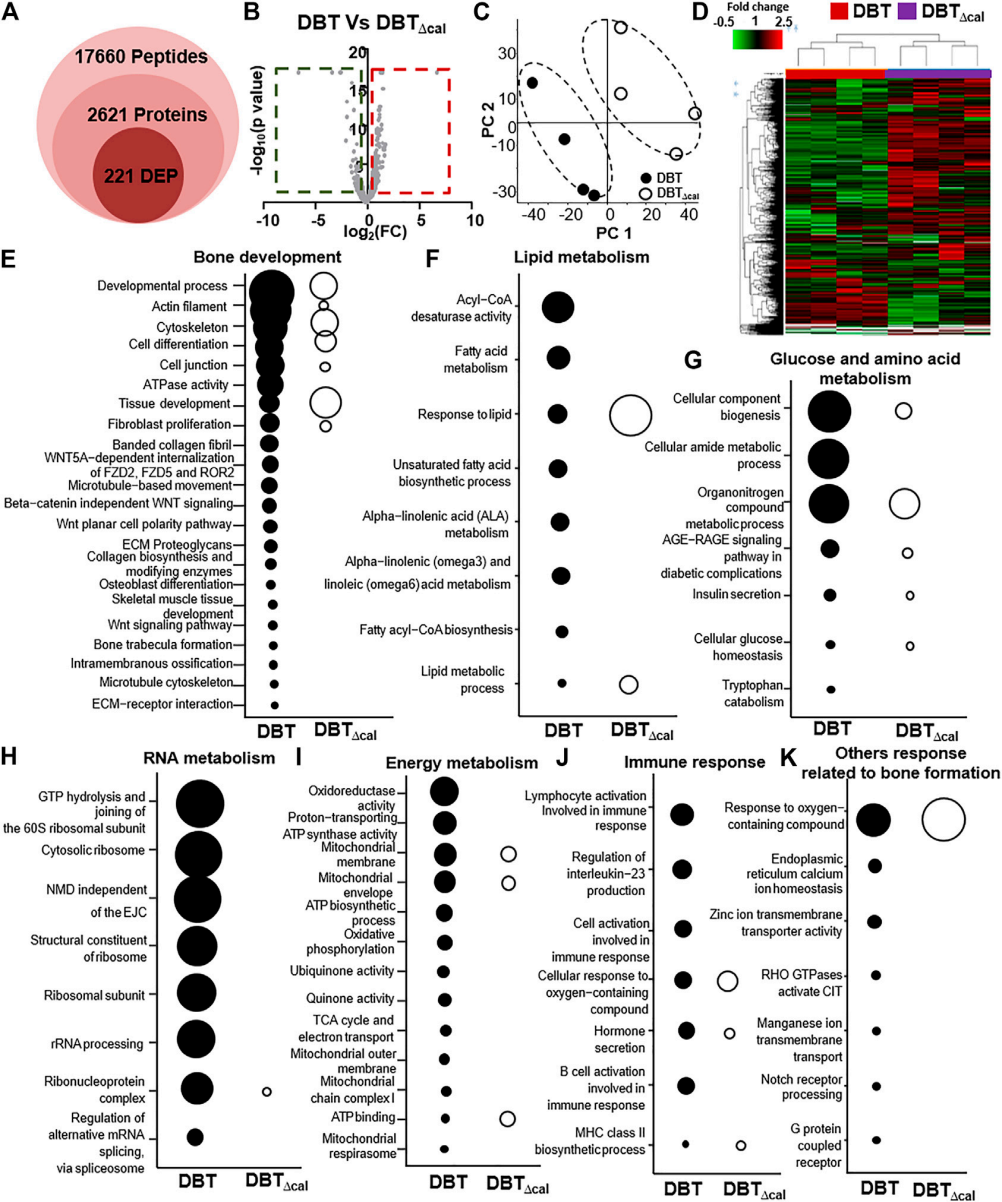
Comparison of proteomics between DBT and DBT_∆cal_. The treatment of osteoblasts was as that in Figure 1. **(A):** The statistical summary of total detected peptide, protein, and DEP. **(B):** In MS results for DBT and DBT_∆cal_, DEPs are deemed significant for *p* < 0.05 and fold change higher/lower than ±1.5, corresponding to the rectangular regions. **(C):** PCA and analyses resolve the observations into two clusters. The components, PC1 and PC2, are reflecting the horizontal and vertical axis, respectively. **(D):** Heat map analysis of identified proteins. The brightness of each color corresponds to the magnitude of difference when compared with average value. Hierarchical clustering is shown on the top of the map. **(E–K):** KOBAS analysis enriches the signaling pathway in DEGs via KEGG, Reactome, BioCye, PANTHER, and GO. The size of the circle represents–log_10_(P).

The proteomics profile of cultured osteoblasts, treated with DBT or DBT_∆cal_, was generated by LC-MS. Label free quantification (LFQ) proteomics by intensity of precursor ion calculation was conducted to reveal the differentially expressed proteins (DEPs) after treating with DBT, as compared with DBT_∆cal_. The overall analysis identified 17,660 peptides mapping to 2,626 proteins that were quantified (Figure 3A). The “volcano plot” of change against *p*-value of LFQ results was constructed: the average spectra number for each comparison was higher than 5 (Figure 3B). We considered a significant differentially expressed protein when *p*-value of *t*-test was <0.05, and the fold change was higher/lower than ±1.5 folds. From the result, 221 proteins (142 up regulated and 79 down regulated) were differentially expressed in DBT-treated osteoblasts, as compared to DBT_∆cal_ group (Figure 3B). To reveal the difference between DBT and DBT_∆cal_ treatment groups. we subjected the normalized protein abundances to principal component analysis (PCA) and heat map clustering. From PCA projection, the maximum variability in the dataset was identified between DBT and DBT_∆cal_ treatments (Figure 3C), having the first component covering 42–53% of data variance. In parallel, the heat map showed similar results as that of PCA, where two major clusters separating the protein abundance were observed (Figure 3D).

After revealing all DEPs, triggered by various herbal extracts, the expected and novel signaling pathways were analyzed by KOBAS 3.0. The signaling important for bone development, e.g., cytoskeleton (*p* = 2.6 × 10^−5^), tissue development (*p* = 6.1 × 10^−6^), osteoblast differentiation (*p* = 2 × 10^−2^) and Wnt signaling pathway (*p* = 2 × 10^−2^), were up regulated after DBT treatment (Figure 3E). This observation was consistent with our previous study reporting the enhancement of osteoblastic function by DBT. In addition, the pathways related to lipid metabolism, e.g., acyl-CoA desaturase activity (*p* = 2.8 × 10^−6^) and fatty acid metabolism (*p* = 1.08 × 10^−4^) (Figure 3F) and the pathways related to glucose and amino acid metabolism, e.g., tryptophan catabolism (*p* = 5 × 10^−2^) and organonitrogen compound metabolic process (*p* = 1.5 × 10^−7^) (Figure 3G), were altered in responding to DBT. Meanwhile, DBT induced a robust RNA metabolism, e.g., proteomes of the structural constituent of ribosome (*p* = 8.98 × 10^−8^), rRNA processing (*p* = 2.65 × 10^−7^) (Figure 3H), and energy metabolism, e.g., oxidoreductase activity (*p* = 8 × 10^−6^), proton-transporting ATP synthase activity (*p* = 7.2 × 10^−5^), mitochondrial membrane (*p* = 9.4 × 10^−5^) (Figure 3I). The immune response was induced by increased proteome of interleukin-23 production (*p* = 4.8 × 10^−4^) and response to interleukin-8 secretion (*p* = 3 × 10^−3^) after DBT treatment (Figure 3J). Furthermore, other novel pathways were up regulated, e.g., response to oxygen-containing compound (*p* = 1.3 × 10^−6^), endoplasmic reticulum calcium ion homeostasis (*p* = 3 × 10^−3^) and G protein-coupled receptor complex (*p* = 4 × 10^−2^) (Figure 3K). In comparing to DBT effects, the induced events of protein regulation, as recognized by proteomics, were not revealed in the herbal treatment of DBT_∆cal_ (Figures 3A–K).

**FIGURE 4:**
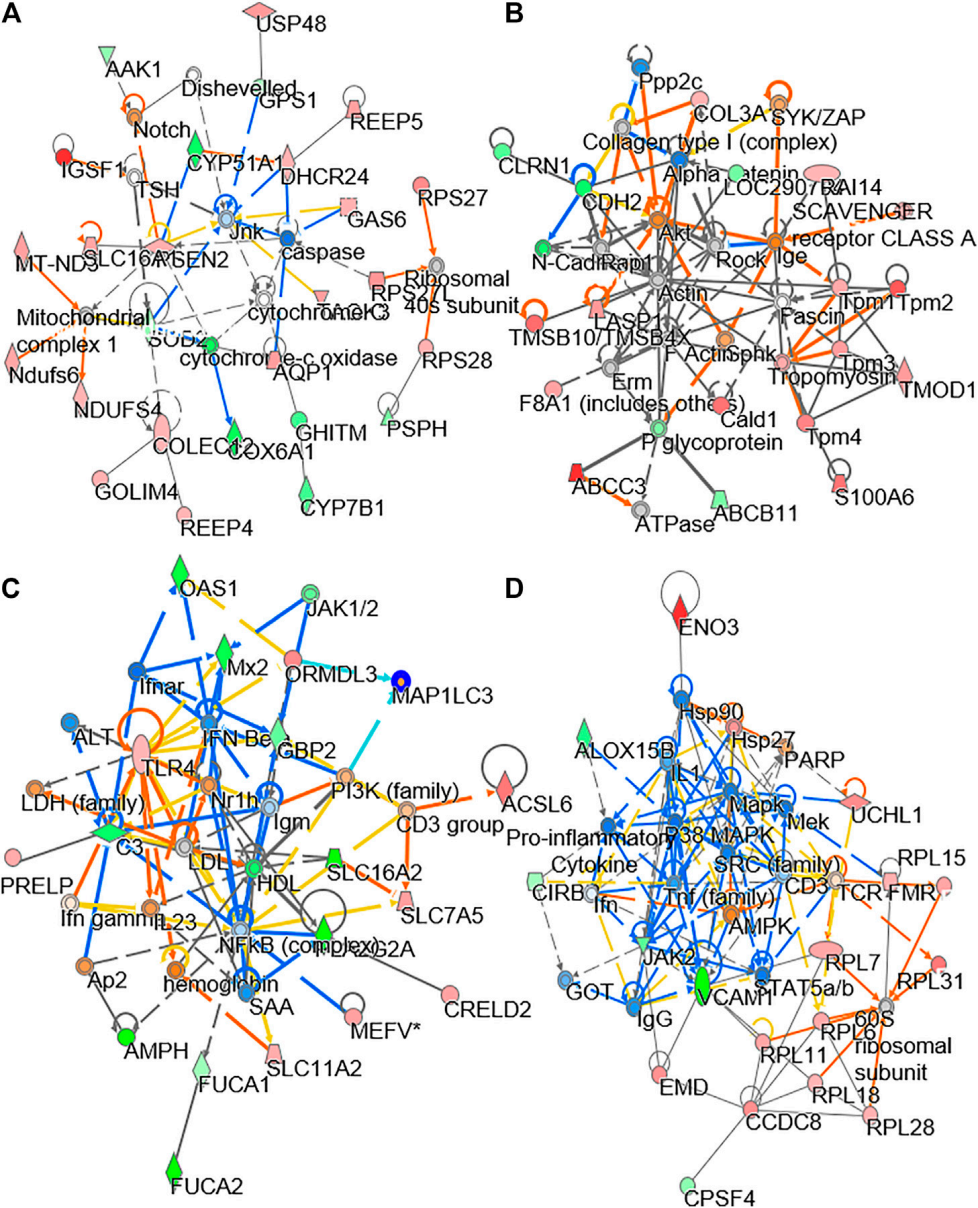
Network analysis of proteomics data. The treatment of osteoblasts was as that in Figure 1. From the proteomics data, the regulated protein in responding to treatments of DBT and DBT_∆cal_ was conducted by molecular network analysis. The network **(A–D)** was obtained by analyzing the DEPs using Ingenuity IPA.

To explore the molecular dynamics of protein-protein interaction from DEPs, the knowledge based-ingenuity pathway analysis and activated molecular prediction *in silico* were constructed. Proteome trajectories were categorized into four significant clusters with up and down regulated DEPs (Figure 4). The first correlation network was centered in inhibiting c-Jun N-terminal kinase (JNK) pathway and activating ribosomal 40S, notch pathway and mitochondrial complex I (Figure 4A). The second one was shown to center in activating Akt, sphingosine kinase-1, (SPHK), tropomyosin, ATPase pathway and inhibiting N-cadherin pathway (Figure 4B). The third one was also centered in inhibiting the NF-KB pathway (Figure 4C), and the fourth one was in activating Figure 4 AMP-activated protein kinase (AMPK), ribosomal 60S, and HSP27 and inhibiting HSP90, inflammation network, SRC and TNF pathways (Figure 4D). These networks are known to be related to bone metabolism, as predicted by an induction of DBT.

### Lipidomics Analysis

**FIGURE 5:**
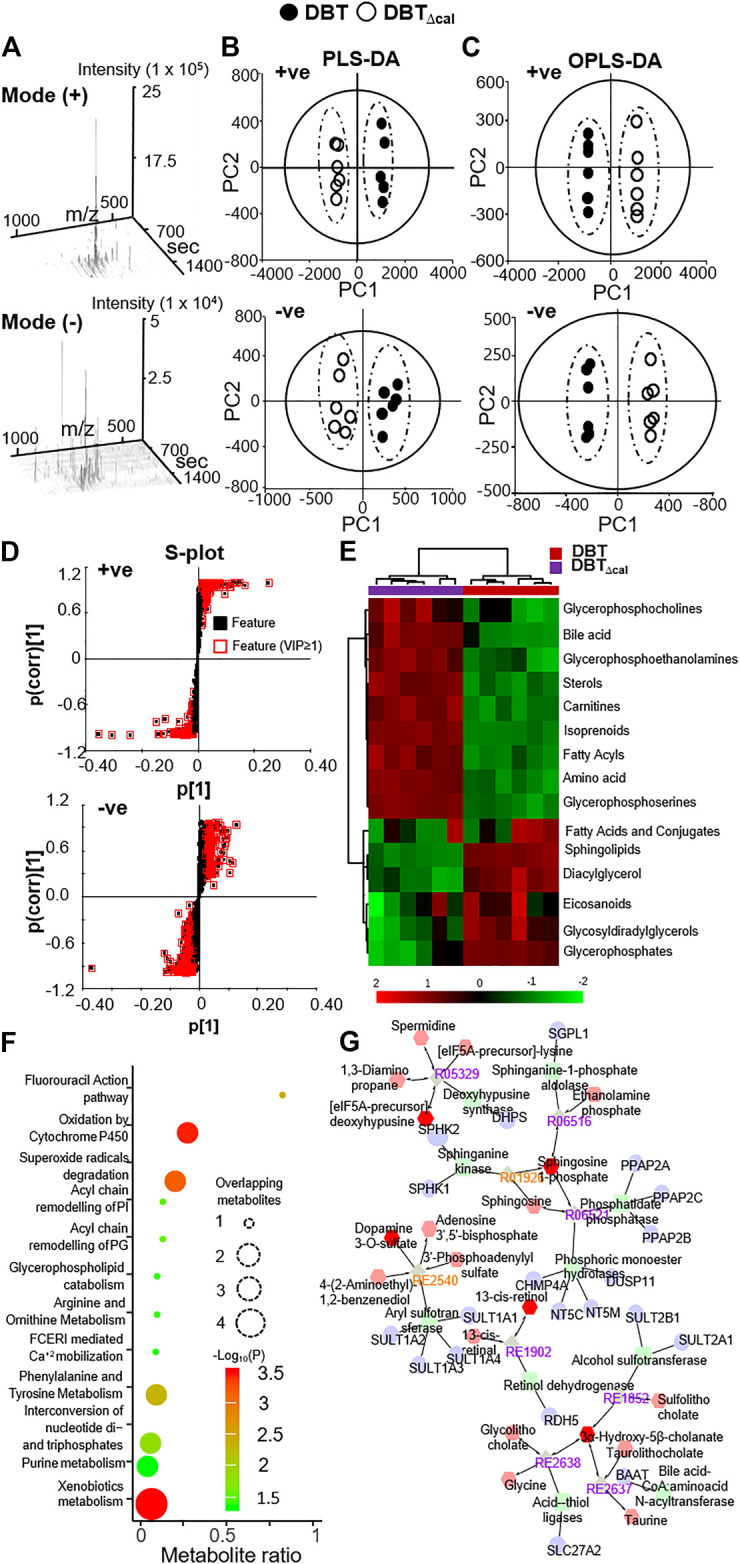
Comparison of lipidomics between DBT and DBT_∆cal_. The treatment of osteoblasts was as that in Figure 1. Lipids were subjected to metabolite analysis by LC-MS. **(A):** The dispersed location of MS/MS scans (+/–) along with retention time, m/z, and intensity shows the optimized LC gradients and efficient separation of the method of acquisition. **(B):** A direct comparison between PLS-DA analyses is identifying two groups of clustering. The components, PC1 and PC2, are reflecting the horizontal and vertical axis, respectively. **(C):** OPLS-DA score plot identifies the maximum separation between DBT and DBT_∆cal_. **(D):** The S-loading plot of OPLS-DA shows the metabolites having a significant difference between DBT and DBT_∆cal_. **(E):** Heat map analysis of identified DEMs. The brightness of each color corresponds to the magnitude of difference when compared with average value. Hierarchical clustering is shown on the top of the map. **(F):** IMPaLA analysis enriched the signaling pathway in DEGs via KEGG. **(G):** Metabolic network analysis from DEMs using Metascape.

In LC-MS analysis, the dispersed location of MS scans along with retention time and m/z illustrated the successful optimized LC gradient and efficient separation of acquisition method, both positive and negative modes (Figure 5A). A total number of features, detected by the cent wave method, was used as a measure of metabolome coverage of this combined strategy. After feature alignment, the total number of 5,611 and 6,690 grouped features were obtained from positive and negative ionization profiles, respectively. In addition, the statistical power was used to find the probability of a real difference of metabolites between experimental groups. After QC filtering, 90% of metabolites were >80% of confidence: these values reflected the difference between groups.

As a quality control procedure, the following steps were routinely performed to ensure system stability and reproducibility of column performance. A formula of standards was injected as QC sample before each experiment. The column pressure was recorded and compared to previous run, and the instrument resolution was optimized for different batches. As shown in Supplementary Figure S2, the QC pool samples were clustered in center of a PCA plot, which suggested that the differentiation of samples was resulted from metabolome difference, but not from systematic variance or technical issues. From multivariate statistical analysis, the fit goodness for PLS-DA and OPLS-DA showed acceptable internal cross-validation results, i.e., partial least squares discriminant analysis (PLS-DA): R^2^X_cum_ = 0.984, R^2^Y = 0.998, Q^2^ = 0.998 in positive ESI mode; R^2^X = 0.964, R^2^Y = 0.994, Q^2^ = 0.99 in negative ESI mode (Figure 5B); and OPLS-DA: R^2^X = 0.987, R^2^Y = 0.99, Q^2^ = 0.99 in positive ESI mode; R^2^X = 0.964, R^2^Y = 0.994, Q^2^ = 0.989 in negative ESI mode (Figure 5C). In score plot of OPLS-DA, the clustering of DBT-treated group was well separated from that of DBT_∆cal_ group (Figure 5C). This indicated that the metabolite profiles of osteoblast at different treatments were very different from each other (Figure 5D). These metabolites and lipids belonged to different classes. The differential expression metabolites (DEMs) were listed in a heat map (Figure 5E). Compared to DBT_∆cal_, the treatment of DBT led to increase of ethanolamine, fatty amide, phosphosphingolipid, quinone and sphingolipid, as well as reduction in acetylcarnitine, glycerophosphocholins (PC), glycerophospho- ethanolamine (PE), glycerophosphoserine (PS) and steroid, significantly. The expected and novel pathways, induced by DBT and DBT_∆cal_, were analysed by IMPaLA (Figure 5F). The *p* value represents the degree of significance in specific pathway perturbation by DEMs. The functions related to drug metabolism, e.g., xenobiotics metabolism (*p* = 3.4 × 10^−4^) and oxidation by cytochrome P450 (*p* = 3.6 × 10^−4^), were detected from DEMs. Moreover, the pathways related to glucose and amino acid metabolism, e.g., phenylalanine and tyrosine metabolism (*p* = 4.65 × 10^−3^) and arginine and ornithine metabolism (*p* = 4 × 10^−2^), were altered in responding to DEMs. Meanwhile, DEMs induced a robust change of lipid metabolism, e.g., glycerophospholipid catabolism (*p* = 4 × 10^−2^), acyl chain remodelling of PI (*p* = 2.93 × 10^−2^) and PG (*p* = 2.93 × 10^−2^).

KEGG pathway analysis *in silico* was employed to reveal the metabolic network. Metabolome trajectories were categorized in three significant clusters with up- and downregulated DEMs (Figure 5G). In cluster 1, the correlation network was centred at the enzyme-compound activity of deoxyhypusine synthase, sphinganine kinase, sphinganine-1-phosphate, phosphatidate and phosphoric monoester hydrolases. In cluster 2, the correlation network was centred at the enzyme-compound activity of alcohol sulfotransferase, CoA: amino acid N-acyltransferase and acid-thiol ligase. In cluster 3, the correlation network was centred at aryl sulfotransferase. In cluster 4, the correlation network was centred at retinol dehydrogenase.

### Integrated Omics Analysis

**FIGURE 6:**
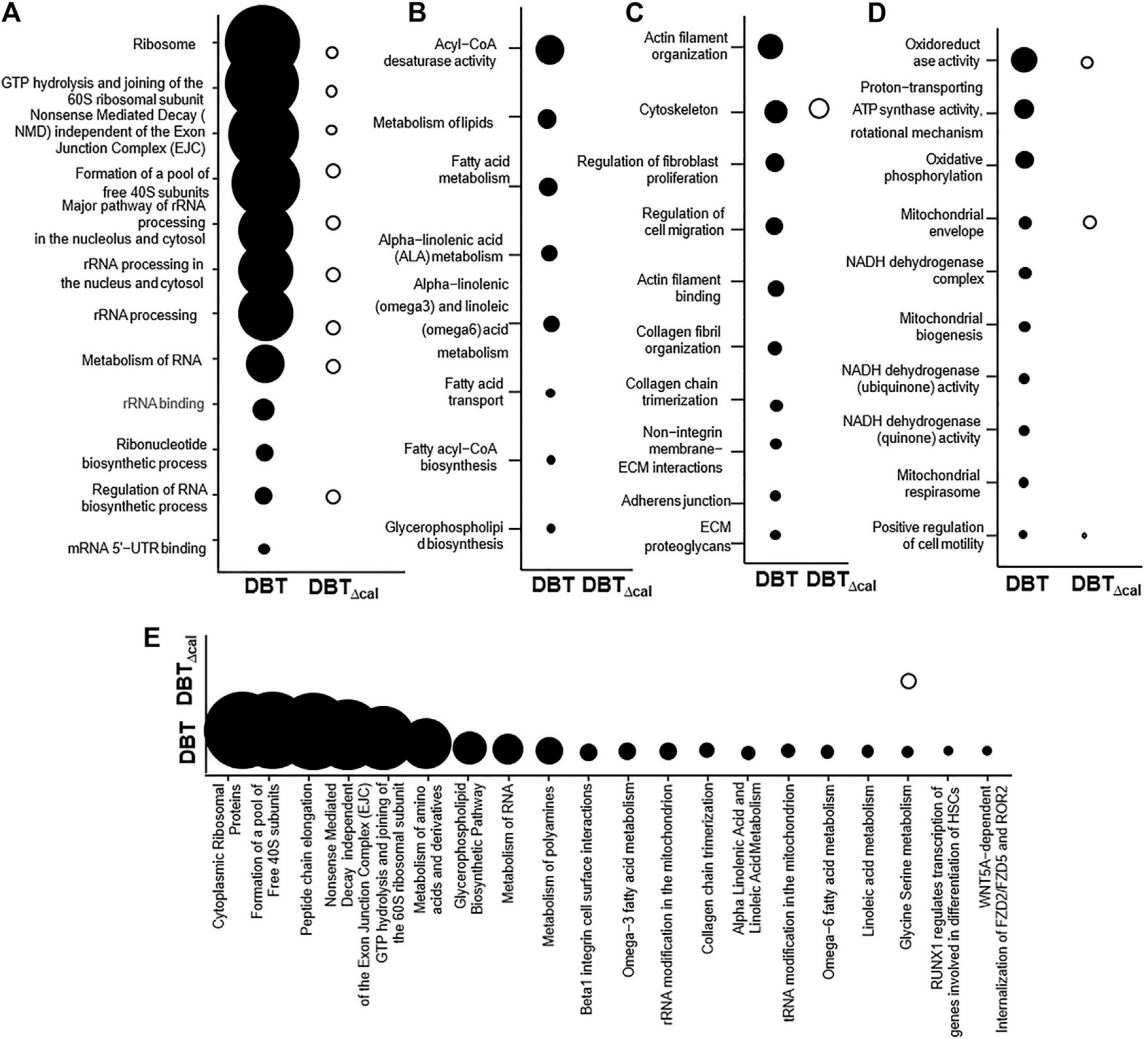
Comparison of integrated omics between DBT and DBT_∆cal_. The treatment of osteoblasts was as that in Figure 1. The transcriptomics, proteomics, and lipidomic data were used. **(A–D):** KOBAS analysis enriches the signaling pathway in DEGs and DEPs via KEGG, Reactome, BioCye, PANTHER and GO. **(E):** IMPaLA analysis enriches the signaling pathway in DEPs and DEMs via KEGG. The size of the circle represents–log_10_(P).

Integrated omics was used to cover the complete biological model by considering different levels of RNA, protein, and lipid regulation. Concatenation-based integration was used in the meta-dimensional analysis (Ritchie et al., 2015). After DBT treatment in contrasting to DBT_∆cal_, the up regulations of pathways relating to RNA metabolism were observed, e.g., small ribosomal subunit GTP hydrolysis and joining of the 60S ribosomal subunit (*p* = 7.4 × 10^−12^) and rRNA processing (*p* = 3.6 × 10^−9^) (Figure 6A), as well as the pathways modulating lipid metabolism, e.g., acyl-CoA desaturase activity (*p* = 3.04 × 10^−5^) and glycerophospholipid biosynthesis (*p* = 6.26 × 10^−6^), were altered in responding to DBT, as compared with DBT_∆cal_. (Figure 6B). In addition, the pathways relating to bone development, e.g., cytoskeleton (*p* = 3.26 × 10^−4^) and actin filament binding (*p* = 1.38 × 10^−4^) (Figure 6C), and energy metabolism, e.g., proton-transporting ATP synthase activity, the rotational mechanism (*p* = 7.68 × 10^−4^) and oxidoreductase activity (*p* = 8.8 × 10^−5^) (Figure 6D), were altered in responding to DBT, as compared with DBT_∆cal_. (Figure 6C). Interestingly, some pathways could be only found in meta-dimensional analysis, e.g., peptide chain elongation (*p* = 5.42 × 10^−10^) and tRNA modification in mitochondrion (*p* = 6.8 × 10^−3^) (Figure 6E).

**FIGURE 7:**
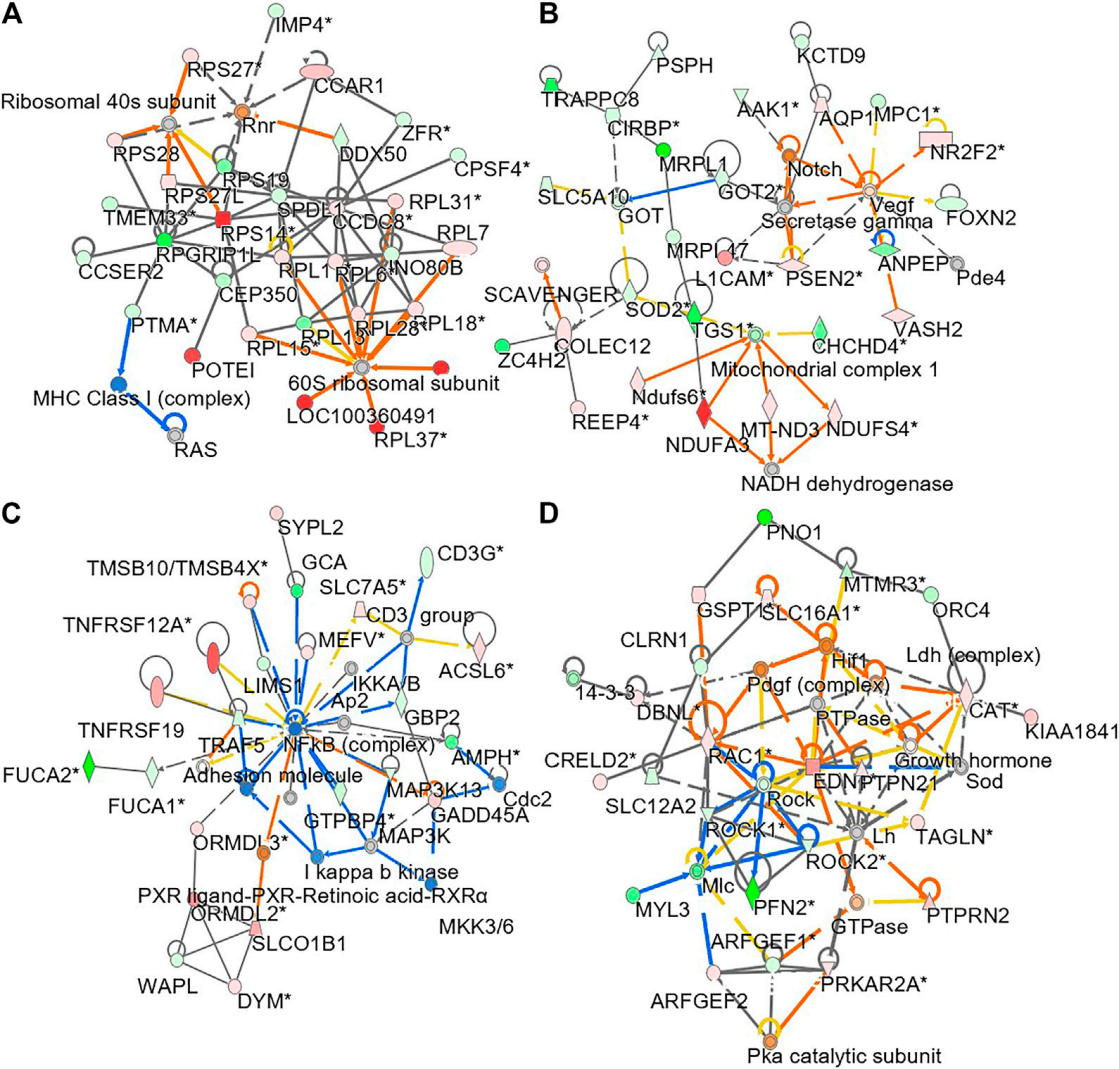
Network analysis of integrated omics data. The treatment of osteoblasts was as that in Figure 1. From the results of transcriptomics, proteomics and lipidomics, the regulated parameter in responding to the treatments of DBT and DBT_∆cal_ was conducted by molecular network analysis. The network **(A–D)** was obtained by analyzing the DEGs, DEPs, and DEMS using Ingenuity IPA.

To understand molecular dynamic from multi-omics data, the knowledge based-ingenuity pathway analysis and molecular activated prediction in silico was employed. Proteome trajectories were categorized in four significant clusters with up- and down-regulated multi-omics data (Figure 7) In cluster 1, the correlation network was centred at activating ribosomal 40 and 60S, which was similar to the previous result in single proteomics level (Figure 7A) In cluster 2, the correlation network was centred at activating VEGF and mitochondrial complex 1 (Figure 7B). In cluster 3, the correlation network was centred at inhibiting NF-kB (Figure 7C). In cluster 4, the correlation network was centred at activating HIF-1α, PDGF and inhibiting ROCK pathway (Figure 7D). In line to the observation, HIF-1α in mature osteoblasts through disruption of von Hippel-Lindau protein is known to profoundly increase angiogenesis and osteogenesis (Wan et al., 2010), and platelet-derived growth factor promotes osteoblast proliferation (Wu et al., 2014). In addition, ROCK activity can trigger cartilage degradation and affect bone formation (Strzelecka-Kiliszek et al., 2017).

## Discussion

The prevalence of age-related diseases is increasing, particularly when life expectancy continues to rise today. The systemic bone disease, e.g., osteoporosis, is a threat to human health: this problem has been reported by half of women aged >50 suffering a risk of osteoporotic fracture. The death rate for patients with a hip fracture is about 24%; so that osteoporosis has been named as “silent killer.” Unfortunately, osteoporosis remains practically incurable at this moment (Rodan and Martin, 2000). One of the most important reasons for this unfavorable situation is poor efficiency of single drug. Under this scenario, the combined drug therapies have been shown to have enhanced effectiveness and minimized side effect (Davis, 2003); however, the action mechanism of combined drugs is remaining unresolved, e.g., the role of individual active compound(s) in a formula. Here, DBT, a Chinese herbal formula containing two herbs (AR and ASR), is an excellent example of combined therapy to demonstrate the requirement of 2 herbs together in activating osteoblastic proliferation and differentiation. The synergy of having AR and ASR at optimized 5:1 ratio has been illustrated by previous reports (Dong et al., 2006; Lin et al., 2017). Furthermore, we are presenting a holistic picture of data science and combined experimental science, as to reveal the final readout of a herbal formula; this omics technology can overcome the key hurdles in revealing action mechanism of combined drugs. The correlation between active chemicals within a herbal formula and its detailed underlying mechanism could be revealed by depleting an interesting bioactive chemical from the formula, as being demonstrated here.

Proper cellular function is relying on careful orchestration of numerous and diverse cellular components, as well as their interactions. The side effect of herbal drug can be viewed as perturbation to an intricate system, either driving it away from homeostasis or aiming to restore the balance. Understanding the orchestrating effect of perturbation is targeting the core of fundamental, as well as practical, challenge in current biological and medical research. Molecular networking provides an unifying and straightforward platform to investigate the orchestrating effect of perturbation of cellular system. In DBT, our previous works have shown the leading orchestrating chemical is calycosin (Gong et al., 2015; Gong A. et al., 2016; Gong A. G. W. et al., 2016; Gong et al., 2018); because the calycosin-depleted DBT cannot induce osteoblastic differentiation, as that of parental DBT. Here, we further illustrate the DBT-induced osteoblastic function does not have much fluctuation in molecular network in comparing to that of calycosin-depleted DBT. Moreover, the results lead credence to the notion that the regulations of transcriptomics, proteomics and metabolomics are greatly influenced by calycosin within DBT, which is in line to its pharmacological functions in cultured osteoblasts (Gong et al., 2018). In the omics analyses, we found that the pathways of proteomics data were richer than that of transcriptomics. In our present study, the results of both omics were generally having a low level of correlation. Thus, we are hypothesizing that the relationship between transcription and translation is likely to vary in expression of individual gene/protein. This may not be realistic to expect a high degree of correlation between RNA and protein levels in an attempt to correlate dynamic change in RNA, e.g., mRNA degradation and non-coding RNA dynamics, with a static picture of the proteome, e.g., collagen and cytoskeleton. This discrepancy is consistent in multi-omics analysis of differentiation of pre-osteoblast cell (Conrads et al., 2005).

Development of bone is focusing on the processes and mechanisms by which “cell number, position, shape, and patterns of connectivity are set during embryonic and early postnatal life.” The proteomic enrichment analyses suggest that the up regulated proteins, triggered by DBT, but not by DBT_∆cal_, are actin filament, cell junction, banded collagen fibril components, ECM-receptor, tropomyosin and Rho GTPase: the regulations of these proteins are in accord to rapid cytoskeleton dynamics and bone regeneration (Dallas and Bonewald, 2010; Huck et al., 2020). In addition, the identified pathways relating to bone differentiation are enriched in the enrichment analysis, in consistent with our previous reports *in vitro* and *in vivo* (Choi et al., 2011; Gong et al., 2015; Zhou et al., 2018; Zhou et al., 2020).

Several lines of evidence suggest the metabolic programming is essential for bone development (Gerstenfeld et al., 1987). Bone progenitor cells primarily depend on glycolysis as their energy source within hypoxic endosteal niches, as well as in favorable oxygen environments (Palomäki et al., 2013). During osteoblastic differentiation, a large amount of extracellular matrix proteins is required to be produced by activated bone cells (Sanchooli, 2019). To support the synthesis of bone matrix, aerobic glycolysis therefore is necessary to provide required intermediates (Langeland, 1975). Besides, the signaling of Wnt and insulin-like growth factor-1 can drive glycolysis during osteoblastic differentiation (Esen et al., 2015). In line to these results, the current omics analysis reveals that the pathway of energy metabolism should be orchestrated under the effect of DBT, e.g., the signaling of insulin receptor, glucose absorption and glycolysis. In supporting this notion, an increase of the grip strength and the swimming time could be revealed in DBT-treated rats, i.e., DBT had a positive influence in promoting aerobic glycolysis (Chang et al., 2020). In addition, the glycolysis flux and capacity were markedly enhanced by DBT in osteoblast (Kwan et al., unpublished data) or cardiomyocyte (Kwan et al., 2019) Moreover, mitochondria play an essential role in bone formation for energy supply. For example, oxidative phosphorylation increases during bone mineralization in responding to ascorbic acid and β-glycerophosphate (Guntur et al., 2014). As a hypothesis, DBT is a potential anabolic agent for clinical disorders of substrate availability-based osteoporosis. Moreover, the current identified pathways have never been identified before in the herbal treatment. For example, the metabolism of amino acids, (e.g. tryptophan and threonine), as induced by DBT, is known to catabolize skeletal muscle cells to produce energy during muscle regeneration (Hocquette et al., 1998).

In lipidomics analysis, the pharmacological properties of DBT were associated with changes of lipids and fatty acids. Lysophosphatidylcholine and phosphatidylcholine can transform to each other, and they are an integral component of cell membrane. Formation of ROS causes the oxidative stress damage and the increase of lysophosphatidylcholine and phosphatidylcholine (Liu et al., 2012), and which thereafter leads to bone loss and friability (Sánchez-Rodríguez et al., 2007). In osteoporotic mice, the content of lysophosphatidyl- choline was significantly up regulated in cell membrane (Zhao et al., 2018). In parallel, an increase of glycerophospholipids was found in osteoporotic mice, suggesting that the abnormal metabolism of glycerolipids and glycerophospholipids could be related to osteoporosis (Zhao et al., 2018). Reduction of glycerolipids by DBT therefore could account for the ROS-mediated osteoporosis. Moreover, sphingolipid was increased in DBT treatment, as shown here. Among these sphingolipids, sphingomyelin is known to regulate cell growth and differentiation, and functions as second messenger (Spiegel and Merrill, 1996).

DBT just depleting one chemical, calycosin, is not able to function as the parental DBT. There are few possible reasons to account for this outcome. First, calycosin may change the membrane structure and facilitate the action of active compounds of DBT. The lateral and rotational freedom of molecules are increased in less ordered membrane. Supporting this notion, sphingomyelin was shown to be induced in DBT, which could induce the distribution of nanodomains in membrane (Krishna et al., 2020). Second, calycosin can collaborate with other compounds in interacting with their corresponding receptors. One possibility is that calycosin may be coupling with formononetin to trigger the receptor for bone differentiation, such as LRP5/6 receptors for Wnt signaling. Third, calycosin is known to stimulate osteogenic differentiation of via activation of insulin-like growth factor-1 receptor signaling and PI3K/Akt signaling (Fang et al., 2019). The signaling of insulin-like growth factor-1 receptor is essential for ossification and bone mineralization (Crane et al., 2013). Moreover, the activation of insulin-like growth factor-1 receptor increases the glycolysis flux and energy metabolism. In addition, 3-kinase/Akt (PI3K/Akt) signaling is involved in osteoblast proliferation and differentiation (McGonnell et al., 2012). This function of calycosin is matching in our omics results. In our future work, the molecular docking must be done in revealing the possible interaction of various protein targets with calycosin, in particular the direct targets in mitochondria being activated by DBT are not known. In addition, the illustration of calycosin in synergy with other phytochemicals within DBT has to be given, even though this may require a large amount of searching activity. The synergy of calycosin with phytochemicals from ASR could be an interesting hypothesis, which may support the formulation of DBT having 2 herbs. Besides, this can support the synergy of AR and ASR in functions of DBT.

## Conclusion

Here, we demonstrated an in-depth and integrated pipeline to reveal the active compound (calycosin) in orchestrating functions of DBT in the cellular system. First, the integration of multi-omics (transcriptomics, proteomics, and lipidomics) enables an exploration of molecular dynamic in different levels of osteoblastic development. Second, by using chemical knockout and integrated omics approach, the osteoblastic function of DBT_∆cal_ is lost relating to glycolysis, energy metabolism, AMPK pathway, lipid metabolism as well as immune response. Third, calycosin must collaborate with other compounds within DBT to maximize the action mechanism of osteoblastic function. As a result, calycosin has a crucial role in controlling interactions with other DBT components in osteoblastic systems precisely, engineeringly and naturally. Our novel approach could serve as a crucial backdrop for future studies that characterize the impact of a broad array of different factors of herbal medicine. In addition, the investigation of action mechanism in applying multi-target drugs, or combinational therapeutics, should be important for the modernization of herbal medicine.

## Data Availability

The datasets presented in this study can be found in online repositories. The names of the repository/repositories and accession number(s) can be found in the article/Supplementary Material.

## Supplementary Material

Supplementary Figure 1 Chemical analysis of DBT and DBT_∆cal._ **(A):** Chemical structure of calycosin. **(B):** Calycosin at different amount was applied onto osteoblast for 7 days to analyze cell proliferation, ALP activity and pRUNX2-Luc response. These parameters were compared between calycosin and vehicle control. **(C)** One hundred mg/mL of herbal extract was subjected to analysis, detected at the wavelength 254 nm. The isolated fraction was shown. **(D)**: ELSD analysis of DBT and DBT_∆cal._ Typical HPLC fingerprint of DBT and DBT_∆cal_ were shown here. One hundred mg/mL of DBT and DBT_∆cal_ was loaded to HPLC column, and the chemical fingerprint was revealed at the ELSD and DAD detector. The isolated fraction was shown. Representative chromatograms were shown, *n* = 4. **(E)**: Quantitative assessment of 5 major chemicals in DBT and DBT_∆cal._ Values are expressed in ng active compound in 1 mg dried extract, in Mean ± SEM, where *n* = 5. ****p* < 0.001 as compared with parental DBT.

Supplementary Figure 2 Multivariate analyses of lipidomic. Lipids from treated osteoblast, as in Figure 1, were subjected to lipidomics analysis by LC-MS in both +ve (left) and -ve (right) modes. A direct comparison between PCA demonstrates that PCA resolves the observation into four clusters. The components PC 1 - 3 are reflecting different axis, as indicated.

## Abbreviations

ALP, Alkaline phosphatase; AR, Astragali Radix; ASR, Angelicae Sinensis Radix; CE, Collision energy; DAD, Diode array detector; DBT, Danggui Buxue Tang; DBT_Δcal_, Calycosin-depleted DBT decoction; DEG, Differentially expressed gene; DEM, Differential expression metabolite; DEP, Differentially expressed protein; DP, Declustering potential; DTT, Dithiothreitol; ELSD, Evaporative light scattering detector; FPKM, Fragment pre kilobase transcriptome per million reads; IAA, Iodoacetamide; IDA, Information-dependent acquisition; LFQ, Label free quantification; MS, MSass spectrometry; MTT, 3-(4,5-dimethylthiazol-2-yl)- 2,5-diphenyltetra- zolium bromide; OPLS-DA, Orthogonal partial least squares discriminant analysis; PCA, Principal component analysis; PLS-DA, Partial least squares discriminant analysis; PC, Glycerophosphocholins; PE, Glycerophosphoethanolamine; PS, glycerophosphoserine; TCM, Traditional Chinese medicine; UPLC, Ultra performance liquid chromatography.
