# Supplementary figures and images for: Integrated Omics Reveals the Orchestrating Role of Calycosin in Danggui Buxue Tang, a Herbal Formula Containing Angelicae Sinensis Radix and Astragali Radix, in Inducing Osteoblastic Differentiation and Proliferation
Source: Front Pharmacol. 2021 Jun 23;12:670947. doi: 10.3389/fphar.2021.670947 (PMC8260986; doi:10.3389/fphar.2021.670947)

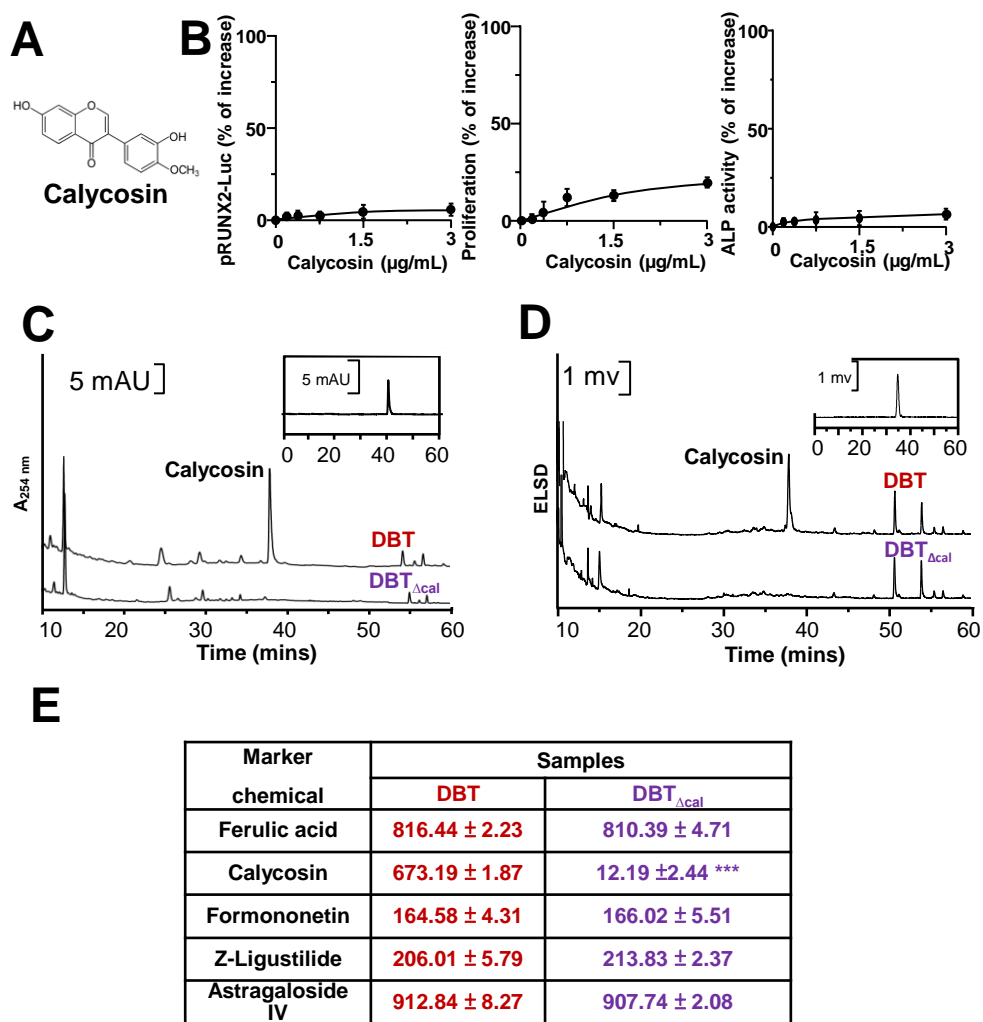

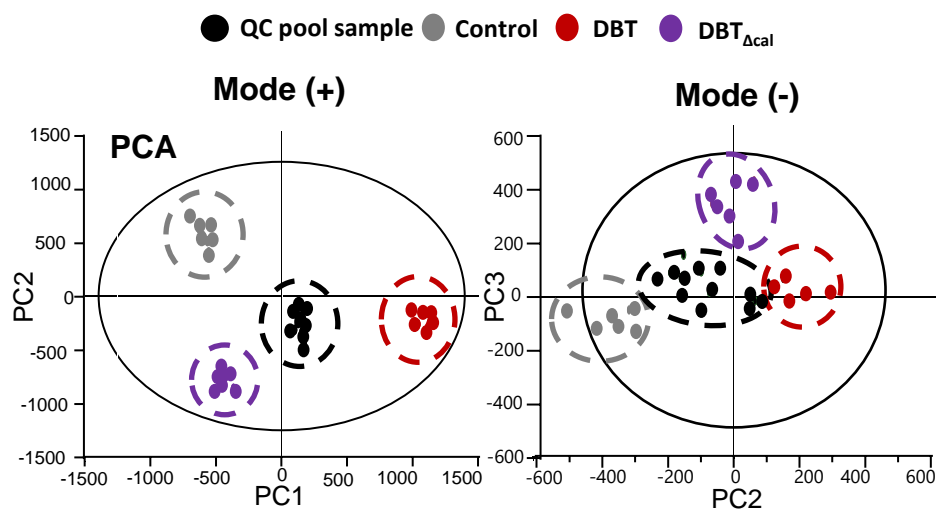

Supplement: Supplementary file 1 [file DataSheet1.PDF]
